# Left atrial cardiomyopathy: association with atrial fibrillation and stroke recurrence

**DOI:** 10.1007/s10554-026-03629-5

**Published:** 2026-01-28

**Authors:** Laura B. H. Friderichsen, Bjørn S. Larsen, Mark Aplin, Nis Høst, Rakin Hadad, Louisa M. Christensen, Hanne Christensen, Inger Havsteen, Litten Bertelsen, Morten A. V. Lund, Thomas Jespersen, Dominik Linz, Ahmad Sajadieh

**Affiliations:** 1 Faculty of Health and Medical Sciences, Department of Biomedical Sciences, University of Copenhagen, SUND, BMI, Blegdamsvej 3B, 2200 København N, Copenhagen, Denmark; 2 Department of Cardiology, Copenhagen University Hospital—Bispebjerg and Frederiksberg, Copenhagen, Denmark; 3 Department of Neurology, Copenhagen University Hospital—Bispebjerg and Frederiksberg, Copenhagen, Denmark; 4 Department of Radiology, Copenhagen University Hospital—Bispebjerg and Frederiksberg, Copenhagen, Denmark; 5 Department of Cardiology, Copenhagen University Hospital—Rigshospitalet, Copenhagen, Denmark; 6 Department of Cardiology, Maastricht University Medical Centre and Cardiovascular Research Institute Maastricht, Maastricht University, Maastricht, The Netherlands

**Keywords:** Atrial cardiomyopathy, Cardiac magnetic resonance, Ischemic stroke, Atrial fibrillation

## Abstract

**Aim:**

To investigate the association between functional and structural markers of left atrial (LA) dysfunction in patients with stroke of either (i) undetermined etiology or (ii) small or large-vessel stroke, and the recurrence of stroke or new-onset atrial fibrillation (AF).

**Methods and results:**

Between 2019 and 2021 we consecutively included 91 patients with a recent stroke (< 30 days) without known or detected AF. All patients had cardiac magnetic resonance with late gadolinium enhancement to determine LA Emptying Fraction (LAEF), LA volumes, and LA fibrosis. Stroke adjudications were performed according to Trial of Org 10,172 in Acute Stroke Treatment classification as either undetermined etiology (*n* = 48) or stroke from large- or small-vessel disease (*n* = 43). The primary endpoint was a composite of stroke recurrence or new-onset AF. During follow-up, fourteen patients (15%) reached the combined primary endpoint of stroke or new-onset AF. Multivariable cause specific regression analysis demonstrated that a lower LAEF was associated with the primary endpoint (Hazard Ratio [HR], 1.41 per 5% decrease [95% CI, 1.09–1.82]) as well as LA enlargement (HR: 1.98 per 5 ml/m2 increase [95% CI 1.11–3.52]). LA fibrosis did not show any associations with the combined endpoint (HR 1.01 [95% CI 0.95–1.07]), or any of its components.

**Conclusion:**

In patients with recent stroke, LAEF and LA enlargement, but not LA fibrosis, are associated with stroke recurrence or new-onset AF. Further studies are required to determine, whether LAEF as a component of ACM is a modifiable risk factor to reduce the risk of recurrent stroke and/or new-onset AF.

**Supplementary Information:**

The online version contains supplementary material available at 10.1007/s10554-026-03629-5.

## Introduction

Despite of vigorous work-up, up to 25% of ischemic strokes remain cryptogenic, i.e. with no identifiable cause [1, 2]. Undiagnosed atrial fibrillation (AF) is often suspected in many of these cases, yet only around 30% of patients demonstrate AF after three years of monitoring from implantable devices [3]. This also applies to patients without stroke but with similar risk factors. Therefore, it is questionable whether AF by itself is the actual cause of index stroke in these patients. Despite guideline-conform work-up [4, 5], the risk of recurrent stroke remains high, especially in cryptogenic stroke patients, where it has been reported to be around 30% during the first year after index stroke [6]. New risk marker for new-onset AF and recurrent stroke in patients with recent stroke is needed.

Atrial Cardiomyopathy (ACM) has been recognized as a potential joint risk factor for both AF and ischemic stroke [7–9]. ACM has been defined as: “Any complex structural, architectural, contractile, or electrophysiological changes affecting the atria with the potential to produce clinically relevant manifestations” [10, 11] Potentially important components of ACM include atrial fibrosis, atrial enlargement, and impaired atrial function, all of which are thought to contribute to AF vulnerability and thus potentially increase the risk of ischemic stroke [8, 10, 12–14]. 

In patients with recent ischemic stroke without known or detected AF, the contribution of markers of ACM to the short-term risk of recurrent stroke and/or new-onset AF remains insufficiently characterized. A better understanding of the relationship between ACM and recurrent stroke and/or new-onset AF may have the potential to identify stroke cause and thereby ultimately present a novel approach to reducing risk of recurrent stroke.

We aimed to explore the association between markers of left atrial (LA) function, specifically LA emptying fraction (LAEF), atrial enlargement and LA fibrosis, and the risk of recurrent stroke or new-onset AF in patients with ischemic stroke without known or detected AF before or during the initial stroke work-up.

## Methods

### Study design

Details of the study design have been published previously [15]. Briefly, the COAST study was a prospective cross-sectional case-control study conducted at Bispebjerg Hospital, a tertiary acute stroke care facility. At this center, the standard stroke evaluation includes a clinical assessment using the NIH Stroke Scale, brain Computed Tomography (CT) with angiography, stroke Magnetic Resonance Imaging (MRI), 12-lead Electrocardiogram (ECG), blood tests, and continuous ECG monitoring. After discharge, all patients undergo 72-hour continuous ECG recording. If they participated in the study, a contrast enhanced Cardiac Magnetic Resonance (CMR) scan, transthoracic echocardiography, additional blood tests within 12 weeks of the initial stroke were acquired.

**Fig. 1:**
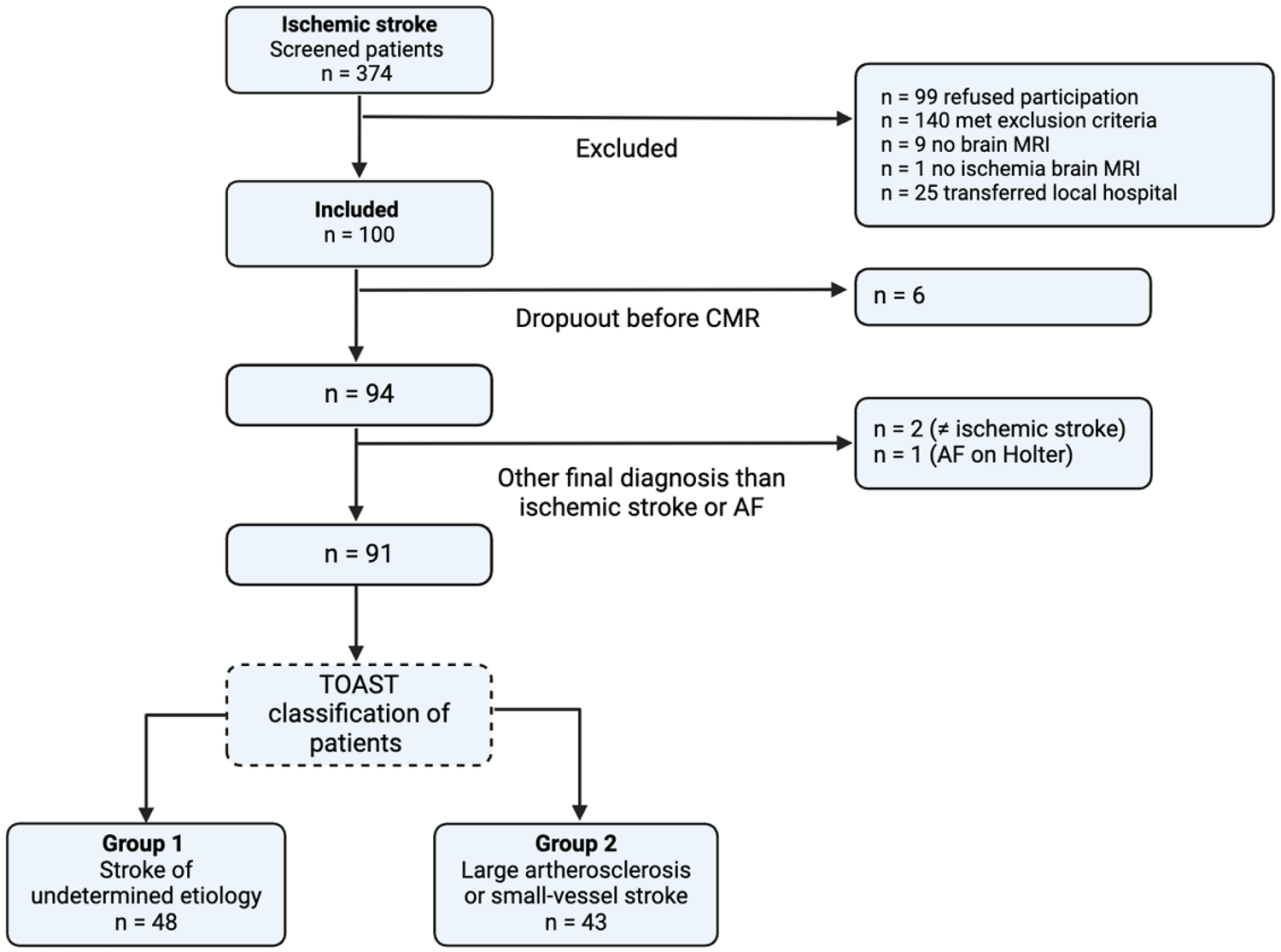
Flowchart of inclusion and classification of patients. MRI, magnetic resonance imaging; CMR, cardiac magnetic resonance imaging; TOAST: Trial of Org 10,172 in Acute Stroke Treatment

### Participants and classification

Enrollment took place between March 12, 2019, and September 6, 2021. During this period, we consecutively included 91 stroke patients, within 30 days of the stroke event. These patients were without known or detected AF during at least 24-hours of continuous monitoring. Further inclusion criteria were age ≥ 18 years and a life expectancy of at least 1 year after enrollment. Flowchart of inclusion and classification of patients is illustrated in Fig. 1. Of the 374 patients screened, a substantial number were excluded based on predefined criteria such as contraindications to CMR, diagnosis of AF, no brain MRI, refusion to participate, or other clinical contraindications, as detailed in Fig. 1. Patients were classified by two board-certified stroke neurologists as either (1) stroke of undetermined etiology or (2) stroke from large-artery atherosclerosis or small-vessel disease. Classification was performed according to the Trial of Org 10,172 in Acute Stroke Treatment (TOAST) criteria [16]. The adjudication was based on all investigations from the clinical workup, ECG recordings, transthoracic echocardiography, and brain imaging analysis. If multiple etiologies were present, the adjudication prioritized the acute ischemic event identified by the diffusion-weighted imaging (DWI) lesion and the CT angiography. Therefore, small cortical or subcortical lesions were not considered indicative of small-vessel disease unless signs of small-vessel disease were found in brain imaging.

### Cardiac magnetic resonance (CMR)

Cardiac MRI was performed using a 1.5T MRI scanner (Magnetom Aera, Siemens Healthcare, Germany) with an 18-channel body coil. The protocol included cine sequences for long-axis (two-, three-, and four-chamber) and short-axis views of the left ventricle (LV), as well as axial cine imaging covering LV. LA late gadolinium enhancement (LA-LGE) imaging was done 20 min after contrast, using a 3D inversion-recovery gradient echo sequence with fat suppression. Scan parameters included a TR/TE of 4.67/1.94 ms, voxel size 1.4 × 1.4 × 2.5 mm³, and images were captured during end-diastole phase of the left atrium to minimize motion. The entire protocol took approximately 45 min.

All CMR scans were analyzed blinded to stroke cause, scan date, and patient information. Volumetric and functional measurements were done by using CVI42 software (v. 5.13.4, Circle Cardiovascular Imaging, Calgary, Canada). Left atrial volumes (LAV) were determined using semiautomated contouring of the LA wall, with manual adjustments based on visual inspection in both two- and four-chamber views. LAV measurements were taken at three time points to evaluate LA function: LAVmax (immediately before mitral valve opening), LAVpreA (just before atrial contraction), and LAVmin (at mitral valve closure). Hereby, total LAEF (was calculated as LAEF = (LAV_max_-LAV_min_)/LAV_max_).

Quantification of LA-LGE was done with the image post-processing software ADAS^®^ (Galgo Medical SL, Barcelona, Spain) as previously described [17]. 

### Follow-up and endpoints

Follow-up was performed in March 2023 from patient records with a median follow-up time of 2.3 years. The primary endpoint was a composite of stroke recurrence or new-onset AF.

The diagnosis of new-onset AF was verified with ECG documentation from electronic patient files and ICD-10 code I48x. A diagnosis of AF outside of a hospital setting in primary care alone was not included. However, in Denmark all patients usually get evaluated in a hospital when AF is detected in primary care. No distinction was made between AF and atrial flutter. The diagnosis of recurrent stroke was based on an ICD-10 code with a new diagnosis of stroke (Ischemic stroke I63x, unspecified stroke I64x) and documented clinical findings consistent with a new stroke. Patients had to have an MRI of the brain with a new lesion described and confirmed by a radiologist. Follow-up rate was 100%. BSL did the adjudication.

### Statistics

Characterization of the cohort regarding clinical variables and demographics was assessed using descriptive statistics. Categorical variables are presented as frequency (%) while continuous variables as mean ± Standard Deviation (SD). LA fibrosis was right skewed with a non-normal distribution and is thus reported with median values and IQR. To estimate the cause-specific hazard of continuous LA parameters, we used multivariable cox regression analysis, adjusting for age, sex, diabetes, and hypertension. Selection of confounding variables was made according to their known relationship with ischemic stroke and AF. A p-value < 0.05 is considered significant. Cumulative incidence curve of the composite endpoint of ischemic stroke and incident AF was performed according to the lowest decile of LAEF. All statistical analysis were performed with R version 4.3.2 (2023-10-31 ucrt).

## Results

### Participants

One hundred patients with recent ischemic stroke were included. 6 patients withdrew before CMR, two patients were excluded as the stroke diagnosis was later refuted, and one had AF detected during initial work-up. In total, 91 patients with stroke were included in the final analysis. Fibrosis calculations are based on data from 78 patients, as 13 scans were excluded due to poor quality of LA-LGE.

**Table 1:**
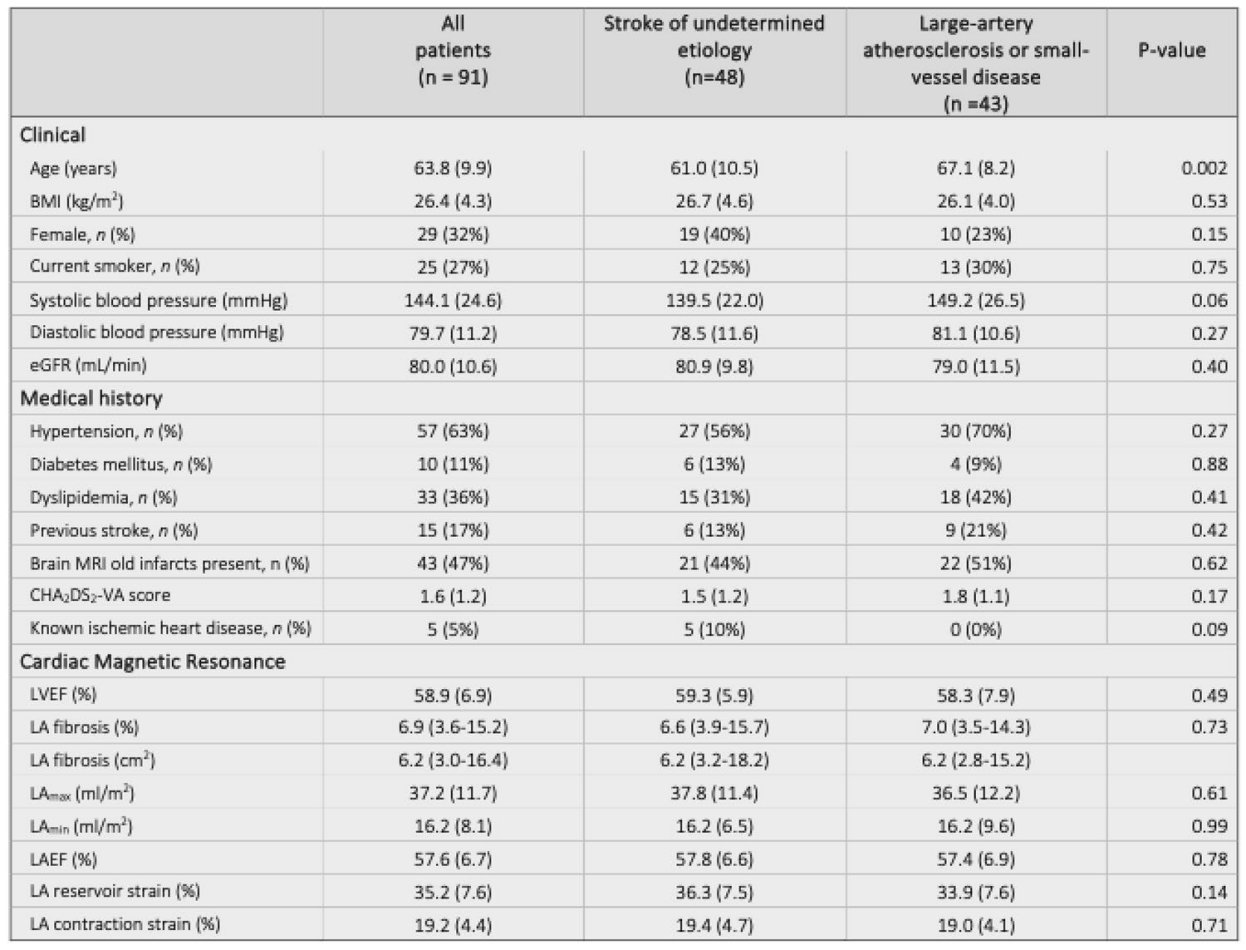
Baseline characteristics of ischemic stroke patients

Values are n(%), mean ± standard deviation, or median (interquartile range). LAEF: left atrial emptying fraction; LA_min_: minimal left atrial volume indexed; LA_max_: maximal left atrial volume indexed; LVEF: left ventricular ejection fraction

### Study population

Baseline characteristics stratified by stroke etiology is presented in Table 1. The patients with large or small vessel disease were significantly older than those with undetermined etiology. The proportion of females were smaller in both groups. All other clinical and demographic parameters showed no significant differences between groups. An additional table presenting baseline characteristics of patients included (*n* = 78) versus those excluded from fibrosis analysis (*n* = 13) is available in the supplement data. This table shows no significant differences between the two groups.

**Table 2:**
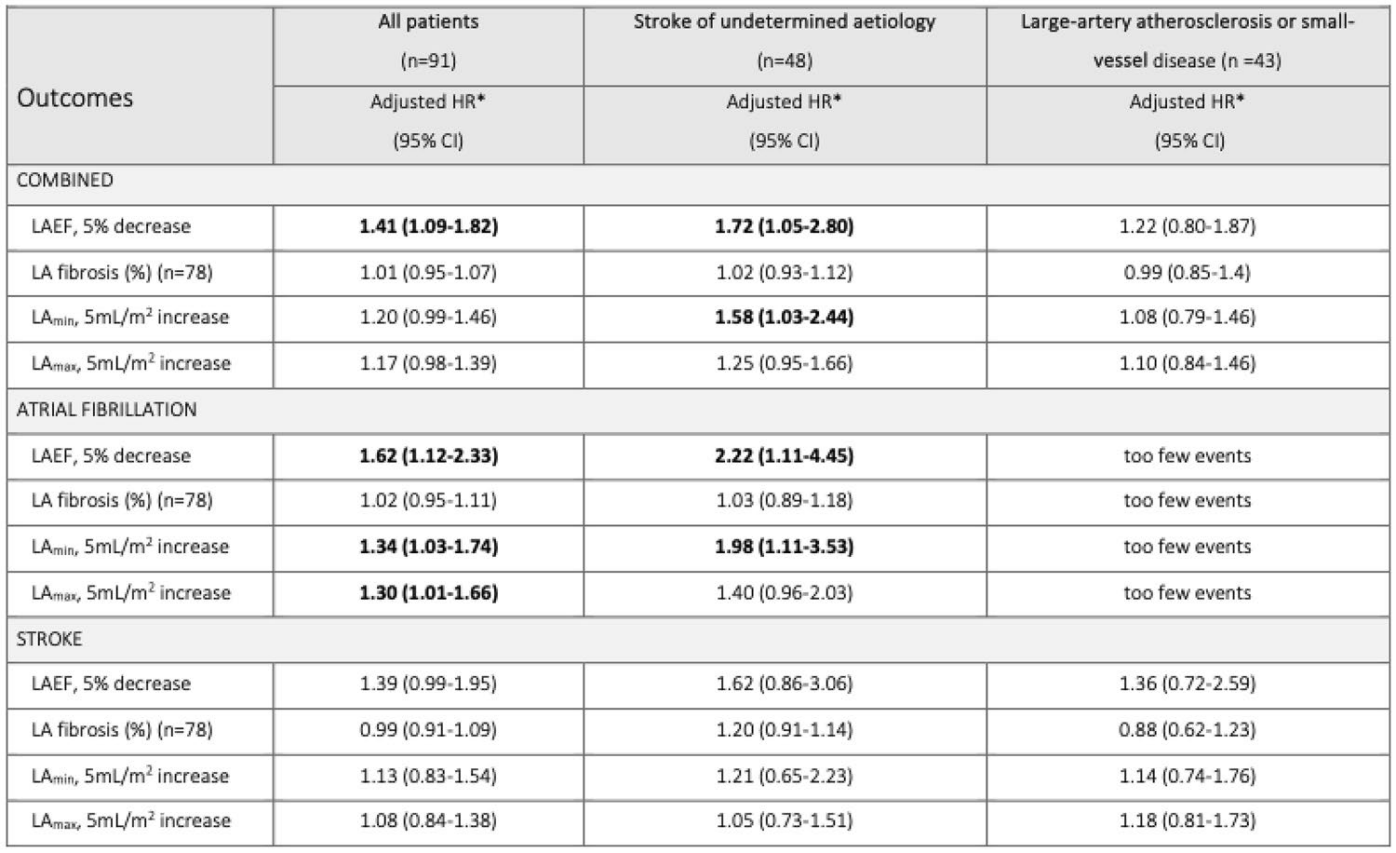
Hazard ratio for ischemic stroke patients and by stroke subtypes

*Adjusted for age, sex, hypertension, and diabetes. LAEF: left atrial emptying fraction; LA_min_: minimal left atrial volume indexed; LA_max_: left atrial maximum volume indexed

### Outcomes

Over a median follow-up of 2.3 years, 14 patients (15%) reached the composite endpoint of recurrent stroke or new-onset AF. Ten patients had a recurrent stroke, seven patients had new-onset AF. Thus, three patients experienced both outcomes. Multivariable cox regression analysis showed that a lower LAEF was associated with the composite endpoint (hazard ratio [HR]: 1.41 per 5% decrease [95% CI, 1.09–1.82]) (Table 2). Subgroup analyses demonstrated a significantly increased risk for patients with undetermined etiology (HR: 1.72 [95% CI 1.05–2.80]), whereas the analysis showed no significant association between LAEF and the composite endpoint for patients with large-artery atherosclerosis or small-vessel disease (Table 2). The analysis did not show any association between LA fibrosis and the composite endpoint (HR: 1.01 [95% CI 0.95–1.07) or the components of the primary endpoint (Table 2). Results regarding LAEF were predominantly driven by a new diagnosis of AF in patients with stroke of undetermined etiology (HR: 2.22 [95% CI 1.11–4.45]), but this could potentially be due to lack of power. Our analysis showed that a decreased LAEF was not significantly associated with the endpoint of recurrent stroke alone (HR: 1.39 [95% CI 0.99–1.95]). Additionally, a significant association between an increase in LA minimum volume and the endpoint new-onset AF was observed (HR: 1.98 per 5 ml/m^2^ increase [95% CI 1.11–3.52]).

**Fig. 2:**
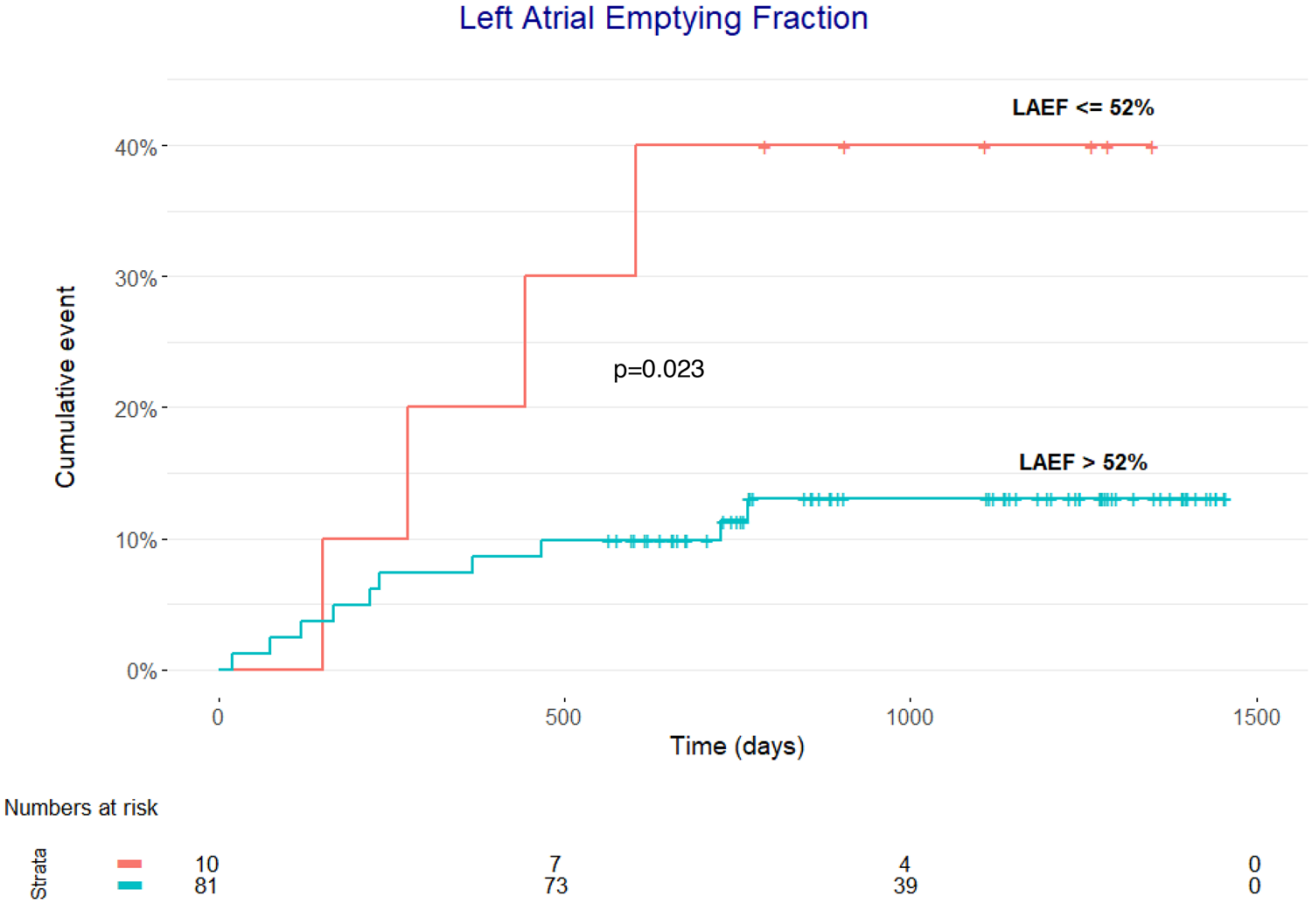
Cumulative incidence curve of composite endpoint according to the lowest decile of LAEF. LAEF; left atrial emptying fraction

Figure 2 shows the cumulative incidence of the composite endpoint, in all patients according to the lowest decile of LAEF (LAEF < 52%). It demonstrates a significant difference according to this cut-off (*p* = 0.023).

## Discussion

In this study we found that a decrease in LAEF, measured using CMR, is associated with a composite endpoint of new-onset AF and recurrence of stroke in patients with recent stroke. We found no significant association between LA fibrosis and the composite endpoint. LA enlargement was associated with increased risk of AF especially in patients with cryptogenic stroke, and minimal LA volume seemed to be more sensitive than maximal LA volume.

### LAEF

Previous studies have shown that LA function, especially total and passive LAEF was associated with incident AF in the general population and device detected AF [18, 19]. Biering-Sørensen et al. found that in patients with undetermined stroke (*n* = 58), the risk of being diagnosed with paroxysmal AF (PAF) was higher for patients with an LAEF in the lowest tertile (LAEF≤ 41%) relative to patients with an LAEF ≥ 50% determined by transthoracic echocardiography (HR: 9.6; [95% CI 1.2–77.3) [20]. In another study of 75 cryptogenic stroke patients, using echocardiographic imaging to detect atrial size and function, a multivariate logistic regression analysis showed an association between detection of AF and LAEF (Odds Ratio: 0.80 [95% CI 0.72–0.89]) [21].

Our findings, along with previous studies, suggest that patients with reduced LAEF may benefit from prolonged ECG monitoring or more frequent follow-up. However, whether AF detected long after the index stroke can be considered its causative factor remains speculative. Additional stroke mechanisms beyond AF may exist, some of which might not be preventable with oral anticoagulation. Nonetheless, identifying clinical AF is highly important as it carries a direct impact on secondary strategies [5]. 

### LA volumes

Several large cohort studies have found that LA enlargement associates with incident AF [22, 23]. Similarly in patients with stroke of undetermined etiology we found a significant association between an increase in minimum volume of the left atrium and the endpoint of new-onset AF (HR 1.98 per 5 ml/m^2^ increase [95% CI 1.11–3.52]). In patients with stroke risk factors but without AF, Bertelsen et al. found similar results in AF incidence [19]. Interestingly, both we and Bertelsen et al. found that only an increase in LA min volume, and not LA max volume, were significantly associated with new-onset AF. This may possibly be due to LA minimum volume being a more sensitive marker to high filling pressure than LA maximum volume. Our findings are consistent with the results of a general population study by Olsen et al. who showed that patients who developed AF (9.4%) had significantly larger LA volumes [18]. 

Several studies suggest that, regardless of AF status, LA enlargement is linked to an increased risk of ischemic stroke [24–26]. With regards to recurrent stroke, Ogata et al. also suggest that LA enlargement is associated with an increased risk of recurrent stroke in AF patients with ischemic stroke (HR: 1.60, [95% CI 1.30–1.98]) [27]. Furthermore, LA enlargement has been found to be associated with an increased risk of recurrent cryptogenic stroke (HR 2.83 [95%CI 1.03–7.81]), but not associated with recurrent total ischemic stroke of all etiologies (HR 1.60 [95% CI 0.48–2.30]) [28]. In contrast, our results showed no significant relationship between LA enlargement and stroke recurrence (LAV_max_ HR 1.08 per 5 ml/m^2^ increase [95% CI 0.84–1.38]; LAV_min_ HR 1.13 per 5 ml/m^2^ increase [95% CI 0.83–1.1.54]). These results were robust when divided into subgroups of stroke etiology.

This is in accordance with recent findings from our group where we found that LAEF but not LA volumes were associated with an increased risk of ischemic stroke in the general population [29]. 

### LA fibrosis

Among the structural changes associated with ACM, LA scarring and fibrosis seems to play a significant role [30, 31]. The extent of native LA fibrosis has been shown to correlate with both the recurrence of AF after ablation [32] and the development of new-onset subclinical AF in patients with implanted loop recorders [11]. Furthermore, there appears to be a bidirectional association between AF and fibrosis with AF promoting further atrial remodeling and fibrosis [4]. 

In regards to stroke subtypes, Fonseca et al. found a significant difference in LA fibrosis percentage between patients with undetermined stroke and those with determined cause (18.5% vs. 10.5%, *p* = 0.03) [33]. Similarly, Kühnlein et al. found that patients with embolic stroke of undetermined source had significantly higher atrial fibrosis compared to lacunar stroke patients (15.0% vs. 10.8, *p* = 0.02) [34]. We found no difference in percentage of LA fibrosis between stroke subtypes (6.6% vs. 7.0%, *p* = 0.73). The difference can perhaps be explained by a lower CHA₂DS₂-VA score, less hypertension, and diabetes of patients in our cohort. We did not find any significant association between LA fibrosis and the endpoint of recurrent stroke (HR 0.99 [95% CI 0.91–1.09]). This might be due to the lack of a prior diagnosis of AF, as this could be a driving factor for both thrombus formation and atrial remodeling. To assess potential bias due to missing data from 13 patients (14.3%) excluded in fibrosis analysis, we compared these clinical outcomes between included and excluded patients. For the ten cases of stroke recurrence, eight of them occurred in the included patient group, and two in the excluded group. For new-onset AF, six of seven events occurred in the included group, and one in the excluded group. Therefore, the distribution of stroke recurrence and new-onset AF was relatively balanced between groups, with event rates of 10.3% vs. 15.4% and 7.7% vs. 7.7%, respectively. These findings suggest that the missing data are unlikely to have introduced systematic bias.

Kühnlein et al. investigated LA fibrosis in patients with embolic stroke of undetermined source, demonstrating that atrial fibrosis (≥ 12%) was associated with an increased risk of recurrent stroke and incident AF (HR: 4.90 [95% CI 1.82–12.66]) in a cohort with a similar sample size and number of events to ours [34]. Despite these similarities, the absence of such associations in our study may be partly explained by the limited number of clinical events, which reduces the statistical power to detect subtle or moderate associations. To our knowledge the study by Kühnlein et al. is the only study that has investigated this association. The discussion is thus still open and awaits results from larger studies.

Furthermore, the discrepancy between our findings and those of Kühnlein et al. may be attributed to differences in fibrosis burden, as their cohort had a mean fibrosis percentage of 15.0% (SD ± 6.2%), whereas our exhibited a mean of 6.6% (IQ 3.9–15.7%). Notably, their findings were statistically significant for fibrosis levels ≥ 12%, a threshold met by only a small subset of our patient population.

### Clinical perspective

AF is well known to increase the risk of ischemic stroke [35–37]. Traditionally, the CHA₂DS₂-VA score is used to assess stroke risk in AF patients. However, recent studies suggests that the underlying burden of AF alone combined with ACM-related structural and functional changes could contribute to an elevated stroke risk [38, 39]. A pooled analysis of individual patient data from five prospective studies found an association between AF burden and ischemic stroke risk (HR 1.03 per hour of AF duration [95% CI 1.00–1.05]) in patients without permanent AF implanted with a device capable of measuring tachyarrhythmia [40]. Yet, it has also been established that decreased LA function, as well as increased LA volume, are associated with a higher incidence of ischemic stroke [41, 42]. This finding may suggest that ACM, rather than AF alone, plays a key role in the pathogenesis of stroke in patients without an established diagnosis of AF [43, 44]. In fact, recent evidence also suggests that ischemic stroke could be the first clinical manifestation of ACM, even without detectable AF [39, 45, 46].

With shared risk factors and underlying pathophysiological mechanisms, it has become clear that the relationship between ACM, AF, and stroke is complex [4]. Therefore, in patients without known AF it may be of crucial importance to understand the specific role of ACM in stroke pathogenesis. While former studies have established links between, on one hand, structural abnormalities, and LA dysfunction, and, on the other hand, a higher risk of ischemic stroke and AF, the immediate risk in stroke patients without pre-diagnosed AF is less well established. Identifying ACM-related markers may allow for better risk stratification and potentially earlier interventions to prevent recurrent stroke or new-onset AF. In this context, our finding that decreased LAEF was predictive of outcomes primarily in patients with undetermined cause of stroke rather than in those with a determined cause, is important. The difference suggests that atrial dysfunction may play a role specifically in cryptogenic stroke, possibly due to silent AF or other undiagnosed atrial abnormalities. It highlights that LAEF may be more useful for identifying risk in this subgroup, rather than in all stroke patients. This distinction underscores the need for tailored approaches to diagnosis and prevention.

Recently, the multicenter randomized trial ARCADIA failed to show efficacy of oral anticoagulation compared to standard antiplatelet treatment in secondary stroke prevention in patients of undetermined stroke etiology with markers of ACM (P-wave terminal force > 5000 mV × ms in ECG lead V1, serum NT-proBNP > 250 pg/mL or LA diameter index ≥ 3 cm/m^2^) [47, 48]. The majority of patients in ARCADIA were included based on high NT-proBNP levels or ECG criteria, which could contribute to the lack of efficacy compared to other risk markers, such as left atrial function, that may be more relevant [47, 48].

These findings highlight the potential importance of determining LA function, particularly LAEF, in assessing the risk of future cardiovascular events in stroke patients. This seems especially relevant in cases with undetermined stroke etiology and suggests that subtype-specific risk stratification could improve clinical decision-making. However, this interpretation remains speculative and should be validated in larger cohorts. Given the potential clinical relevance of LAEF it is essential to consider the imaging modalities used to assess LA function and structure. This study focuses on LAEF and LA volumes measured by CMR, which is recognized as the gold standard [49]. According to the literature, there is a strong correlation between LAEF measured by echocardiography and CMR [50]. As echocardiography is already a standard procedure for the majority of stroke patients, the feasibility of measuring LAEF in routine clinical settings is high. The comparison of CMR and echocardiography is an interesting and requires dedicated investigation.

Future studies are needed to determine the best treatment strategy beyond intensified stroke and AF risk factor modification in this challenging population to improve outcomes.

### Strengths and limitations

This was a prospective cohort-study building on data from the COAST study which was performed with a sample size according to the prespecified calculation. Methods and procedures were preemptively described and published, including a detailed data analysis plan for follow-up, comprising a hazard model to evaluate the combined endpoint of stroke recurrence and atrial fibrillation.

We recognize that our study has limitations. This is an exploratory analysis, and the observational design and modest sample size limit the ability to draw causal claims. Although we adjusted for age, sex, diabetes, and hypertension, residual confounding cannot be excluded. Unmeasured cardiovascular risk factors such as subclinical coronary artery disease, heart failure with preserved ejection fraction, and systemic inflammation may contribute to both LA dysfunction and adverse outcomes.

Furthermore, there is no established agreement of LA-LGE imaging or post-processing methods to quantify LA-LGE, and there is a lack of consensus for grading LA fibrosis. Limitations due to different scanner field strengths (1.5 T vs. 3 T), contrast-related factors and compliance during scanning, affects the quality of images and thus detection of LA-LGE. Other limitations regarding detection of LA-LGE has been described earlier [17].

Although fibrosis data were missing in 13 out of 91 patients (14.3%), a comparison of baseline characteristics between patients included versus excluded in fibrosis analysis showed no significant differences. Moreover, the distribution of stroke recurrence and new-onset AF was relatively balanced between groups, and none of the excluded patients had renal dysfunction or known arrhythmias. These findings reduce the likelihood of systemic bias affecting the fibrosis-related outcomes.

The participants comprising the study population were relatively young, and the follow-up timeframe was short, which impact the number of outcomes. On the other hand, the short follow-up makes the possible biological association more likely. The COAST study was performed in a predominantly Caucasian population, including patients who were able to give consent, and this carries potential to exclude more severe stroke cases, thus narrowing the ischemic stroke spectrum.

## Conclusion

Our findings suggest that in patients with recent stroke without pre-diagnosed AF, LAEF may serve as a risk marker of new-onset AF and stroke recurrence. In this study, we did not find any correlation between the risk of stroke recurrence or new-onset AF and LA fibrosis.

## Supplementary Information

Below is the link to the electronic supplementary material.

Supplementary Material 1

Supplementary Material 2

## Data Availability

The data that support the findings of this study are not openly available due to reasons of sensitivity and are available from the corresponding author upon reasonable request.

## Declarations

### Clinical Trial Number

This study is a follow-up analysis of participants originally enrolled in the COAST study, registered at ClinicalTrials.gov (NCT03830983).
