# Supplementary material for: Left atrial cardiomyopathy: association with atrial fibrillation and stroke recurrence
Source: Int J Cardiovasc Imaging. 2026 Jan 28;42(5):835–44. doi: 10.1007/s10554-026-03629-5 (PMC13136217; doi:10.1007/s10554-026-03629-5)
Supplement: Supplementary file 1 — Supplementary Material 1 [file 10554_2026_3629_MOESM1_ESM.pdf]

Table 3 Baseline characteristics of ischemic stroke patients included vs excluded in fibrosis analysis

	All patients (n = 91)	Included in fibrosis analysis (n=78)	Excluded in fibrosis analysis (n =13)	p-value
**Clinical**				
Age (years)	63.8 (9.9)	64.4 (9.4)	61.0 (12.8)	0.38
BMI (kg/m^2)	26.4 (4.3)	26.5 (4.3)	25.6 (4.3)	0.49
Female, *n* (%)	29 (32%)	28 (36%)	1 (8%)	0.09
Current smoker, *n* (%)	25 (27%)	19 (24%)	6 (46%)	0.20
Systolic blood pressure (mmHg)	144.1 (24.6)	145.1 (25.4)	138.0 (18.7)	0.25
Diastolic blood pressure (mmHg)	79.7 (11.2)	79.9 (11.1)	78.8 (11.8)	0.76
eGFR (mL/min)	80.0 (10.6)	80.2 (10.9)	78.5 (8.6)	0.52
**Medical history**				
Hypertension, *n* (%)	57 (63%)	49 (63%)	8 (62%)	1
Diabetes mellitus, *n* (%)	10 (11%)	8 (10%)	2 (15%)	0.95
Dyslipidemia, *n* (%)	33 (36%)	26 (67%)	7 (53%)	0.27
Previous stroke, *n* (%)	15 (17%)	15 (19%)	0 (0%)	0.19
Brain MRI old infarcts present, n (%)	43 (47%)	34 (44%)	9 (69%)	0.16
CHA_2DS_2-VA score	1.6 (1.2)	1.6 (1.2)	1.6 (1.8)	0.94
Known ischemic heart disease, *n* (%)	5 (5%)	4 (5%)	1 (8%)	1
**Cardiac Magnetic Resonance**				
LVEF (%)	58.9 (6.9)	59.3 (1.4)	56.5 (6.7)	0.27
LA_{max} (ml/m^2)	37.2 (11.7)	37.2 (11.9)	37.1 (11.5)	0.61
LA_{min} (ml/m^2)	16.2 (8.1)	16.3 (8.5)	15.4 (5.5)	0.62
LAEF (%)	57.6 (6.7)	57.4 (7.1)	58.9 (3.9)	0.26
LA reservoir strain (%)	35.2 (7.6)	35.0 (7.4)	36.4 (8.9)	0.61
LA contraction strain (%)	19.2 (4.4)	19.2 (4.4)	19.5 (4.5)	0.79

Values are n (%), mean ± standard deviation, or median (interquartile range). LAEF: left atrial emptying fraction; LA_{min}: minimal left atrial volume indexed; LA_{max}: maximal left atrial volume indexed; LVEF: left ventricular ejection fraction
