# Supplementary material for: Left atrial cardiomyopathy: association with atrial fibrillation and stroke recurrence
Source: Int J Cardiovasc Imaging. 2026 Jan 28;42(5):835–44. doi: 10.1007/s10554-026-03629-5 (PMC13136217; doi:10.1007/s10554-026-03629-5)
Supplement: Supplementary file 2 — Supplementary Material 2 [file 10554_2026_3629_MOESM2_ESM.pdf]

Table 4 Baseline characteristics of ischemic stroke patients excluded in fibrosis analysis

	All patients (n = 13)	Stroke of undetermined etiology (n=6)	Large-artery atherosclerosis or small-vessel disease (n =7)	p-value
**Clinical**				
Age (years)	61.0 (12.8)	55.3 (6.7)	65.8 (15.2)	0.13
BMI (kg/m^2)	25.6 (4.3)	26.2 (3.1)	25.1 (5.2)	0.63
Female, *n* (%)	1 (8%)	0 (0%)	1 (14%)	1
Current smoker, *n* (%)	6 (46%)	3 (50%)	3 (43%)	1
Systolic blood pressure (mmHg)	138.0 (18.7)	141.0 (24.4)	135.4 (13.8)	0.63
Diastolic blood pressure (mmHg)	78.8 (11.8)	81.3 (16.6)	76.5 (6.3)	0.53
eGFR (mL/min)	78.5 (8.6)	81.3 (5.1)	76.0 (10.6)	0.27
**Medical history**				
Hypertension, *n* (%)	8 (62%)	3 (50%)	5 (71%)	0.83
Diabetes mellitus, *n* (%)	2 (15%)	2 (33%)	0 (0%)	0.37
Dyslipidemia, *n* (%)	7 (53%)	3 (50%)	4 (57%)	1
Previous stroke, *n* (%)	0 (0%)	0 (0%)	0 (0%)	1
Brain MRI old infarcts present, n (%)	9 (69%)	4 (67%)	5 (71%)	1
CHA_2DS_2-VA score	1.6 (1.8)	1.3 (1.0)	1.8 (1.3)	0.44
Known ischemic heart disease, *n* (%)	1 (8%)	1 (17%)	0 (0%)	0.94
**Cardiac Magnetic Resonance**				
LVEF (%)	56.5 (8.1)	57.4 (4.2)	55.8 (10.7)	0.72
LA_{max} (ml/m^2)	37.1 (11.5)	34.4 (12.5)	39.4 (11.0)	0.46
LA_{min} (ml/m^2)	15.4 (5.5)	14.0 (5.2)	16.6 (6.0)	0.42
LAEF (%)	59.1 (3.9)	59.3 (2.6)	58.7 (5.0)	0.76
LA reservoir strain (%)	36.4 (9.1)	37.5 (7.6)	35.4 (10.6)	0.69
LA contraction strain (%)	19.5 (4.5)	20.8 (4.9)	18.4 (4.1)	0.38

Values are n (%), mean ± standard deviation, or median (interquartile range). LAEF: left atrial emptying fraction; LA_{min}: minimal left atrial volume indexed; LA_{max}: maximal left atrial volume indexed; LVEF: left ventricular ejection fraction
